# Correlates of preconception and pregnancy hair cortisol concentrations

**DOI:** 10.21203/rs.3.rs-3349003/v1

**Published:** 2023-09-21

**Authors:** Diana L. Juvinao-Quintero, Richard G. Künzel, Gloria Larabure-Torrealva, Laramie Duncan, Clemens Kirschbaum, Sixto E. Sanchez, Bizu Gelaye

**Affiliations:** Department of Epidemiology, Harvard T.H. Chan School of Public Health; Department of Epidemiology, Harvard T.H. Chan School of Public Health; Instituto Nacional Materno Perinatal; Department of Psychiatry and Behavioral Sciences, Stanford University; Technische Universität Dresden; Universidad de San Martin de Porres, Facultad de Medicina Humana, Instituto de Investigacion; Department of Epidemiology, Harvard T.H. Chan School of Public Health

**Keywords:** hair cortisol concentrations, preconception, pregnancy, modifiable risk factors, correlates

## Abstract

Assessing factors that influence chronic stress biomarkers like hair cortisol concentrations (HCCs) in pregnancy is critical to prevent adverse pregnancy outcomes. Thus, we aimed to identify correlates of HCC preconception and during pregnancy. 2,581 pregnant women participated in the study. HCC was available at four time periods: pre-pregnancy (0–3 months preconception, n = 1,023), and in the first (1–12 weeks, n = 1,734), second (13–24 weeks, n = 1,534), and third (25–36 weeks, n = 835) trimesters. HCC was assessed using liquid chromatography tandem mass spectrometry (LC-MS/MS). Sociodemographic, pregnancy- and hair-related characteristics, and measures of psychosocial stress, were interrogated as potential correlates of HCC. Spearman correlations, paired t-tests, and ANOVA were used to assess differences in log-transformed values of HCC (logHCC) across maternal characteristics. Multivariable linear regressions were used to identify the correlates of HCCs after adjusting for confounders. Mean logHCC values increased across the four prenatal periods (*P* < 0.001). In multivariable analyses, pre-pregnancy BMI was consistently associated with all HCCs, while gestational age, economic hardship, hair dyeing, and depression, showed time-specific associations with HCC. In conclusion, this study showed evidence of factors influencing HCC levels before and during pregnancy. The most consistent association was seen with pre-pregnancy BMI. Depression was also associated with HCC concentrations.

## INTRODUCTION

Psychological distress is one of the most pressing issues of public health worldwide. Globally, more than 250 million people are affected by depression ^1^ and more than 45 million people suffer from anxiety, making both disorders the leading contributors to global burden of disease and global disability ^1–4^. Women are disproportionally more exposed to stressors and factors that contribute to depression and anxiety than men ^4,5^, particularly in women of reproductive age and from low- and middle-income countries (LMICs) ^5,6^. In LMICs, symptoms of antenatal psychological distress, including depression and anxiety, are often unrecognized and untreated ^7^, and are associated with an elevated risk for preterm birth ^8–12^, low birth weight ^13^, postpartum depression ^14–16^, and impaired infant neurodevelopment ^17,18^ among others, implicating adverse health outcomes across generations ^8^.

The biological mechanisms linking antenatal distress to adverse health outcomes remain incompletely understood, partly because the specific etiology of antenatal distress, such as depression and anxiety itself is highly multifaceted ^19–21^. One facet that contributes to anxiety and depression etiology is the experience of severe or chronic stress ^22^. A well-understood biological stress response system is the hypothalamic-pituitary-adrenal (HPA) axis, which becomes increasingly activated during stressful experiences, leading to the secretion of glucocorticoids and catecholamines ^23–25^. The effector glucocorticoid of the HPA axis is cortisol, which can be used as an objective biomarker of stress ^23–25^. Due to situational and diurnal changes in cortisol secretion, cortisol measurement is challenging ^26^. Responding to these challenges, cortisol measurement in hair emerged as a promising biomarker, because it provides retrospective, aggregate measurement of cortisol secretion over long time periods (e.g. up to 9 months) ^27,28^.

Interestingly, HPA axis glucocorticoids also undergo massive alterations during the pregnancy course ^29–32^, indicating that HPA axis activity may be a decisive correlate to the elevated mental disorder prevalence of pregnant women. However, previous studies investigating the association between hair cortisol concentration (HCC) and antenatal psychological distress yielded inconclusive results showing both, positive associations ^33,34^ and null findings ^35–37^. Some of these studies have only poorly or highly heterogeneously addressed the influence of pregnancy-related and hair-related characteristics on HCC ^26^, despite the importance of characterizing HCC correlates to understand its influence on maternal mental health and pregnancy outcomes. Lastly, sample sizes in previous studies have been rather small (N range 23 to 768, median N = 108), and may not have had adequate statistical power for detecting the effects investigated ^37–41^. Given (i), the intergenerational impact on health outcomes associated with antenatal mental health disorders, particularly in LMICs, and (ii) inconclusive prior results about pregnancy-related, characteristics, and hair-related HCC correlates, we investigated (a) the influence of pregnancy and hair-related characteristics on HCC levels before and throughout pregnancy, and (b) the association between prenatal HCC and symptoms of antenatal depression, anxiety, and stress, using a large sample of pregnant Peruvian women (N = 2,581).

## RESULTS

### Population at baseline

We described the characteristics of the study population in Table 1. Briefly, the mean ± SD age of participants was 28 ± 6.3 years, most of them identified themselves as mestizo (84%), were married (83%), had normal (18.5–24.9 kg/m^2^) pre-pregnancy BMI (48%) and were multiparous (56%). Half of the participants reported having more than 12 years of education, almost the same proportion (47%) was employed in pregnancy, 39% reported having difficulty accessing basic foods, and 57% indicated that the index pregnancy was unplanned. Only 2% and 8% of participants reported tobacco smoking and using alcohol during pregnancy, respectively. Antenatal symptoms of depression and anxiety showed a prevalence of 23% and 48% in our study sample, respectively, and the mean PSS value was 19.9 ± 7.38.

### Differences between HCCs

We observed increasing values of HCC from the pre-pregnancy period to the third trimester (Fig. 2). For instance, mean values of HCC pre-pregnancy (mean = 3.8 ± *SD* = 4.1 pg/mg) were 0.15 logHCC units lower (*n* = 1022, 95%CI = 0.11, 0.19) than mean values of HCC in the first trimester (4.1 ± 3.9 pg/mg). Mean values of HCC in the second trimester (4.5 ± 5.0 pg/mg) were 0.21 logHCC units higher (*n* = 694, 95%CI = 0.16, 0.25) than mean values of HCC in the first trimester. Lastly, mean values of HCC in the third trimester (5.6 ± 4.4 pg/mg) were 0.35 logHCC units higher (*n* = 832, 95%CI = 0.32, 039) than *mean* values of HCC in the second trimester. Using the Pearson’s method, strong positive correlations (r range 0.62–0.70, *P* < 0.001) were seen in the pairwise comparisons of logHCCs between the four consecutive periods (Supplementary Table 1).

### Bivariate analysis

In the bivariate analysis, pre-pregnancy BMI was positively associated with logHCCs in all time periods (r range 0.11 to 0.18, *P* < 0.001). Gestational age at HCC collection was negatively associated with logHCC in the first and second trimesters (r range − 0.064 to −0.065, *P* < 0.01), while a positive association was seen with logHCC in the third trimester (r = 0.15, *P* < 0.001) (Table 2). Parity was correlated with logHCC values pre-pregnancy (r = 0.10, *P* < 0.001) and in the third trimester (r = −0.09, *P* < 0.01), and the PSS was inversely correlated with logHCC in the second trimester (r = −0.02, *P* < 0.01). GAD-7 and the PHQ-9 scores showed a tendency towards an association with logHCC in the second (r = −0.05) and third trimester (r = 0.07) (*P* ≤ 0.05). Including adjustment variables (maternal age, gestational age, pre-pregnancy BMI, and parity) did not change the magnitude of these correlations substantially (Table 2). Statistically significant differences in the geometric mean of HCCs were also seen with respect to categories of maternal age, pre-pregnancy BMI, parity, education, employment, difficulty accessing basic foods, infant sex, hair dyeing, frequency of hair cutting, and GAD-7 in at least two time periods (Table 3). As shown in Fig. 3, a trend towards increasing values of logHCCs was seen with increasing values of pre-pregnancy BMI in all time periods (*P*_*ANOVA*_ < 0.01). LogHCCs in the first and second trimesters decreased with increasing years of education (*P*_*ANOVA*_ < 0.001), and a U-shape association was seen with maternal age (*P*_*ANOVA*_ < 0.05) in the same trimesters. Having difficulty accessing basic foods, dyed hair, and a female infant, were commonly associated with higher logHCC in the pre-pregnancy and pregnancy periods (Fig. 3). Participants with symptoms of antenatal anxiety (GAD-7 score ≥ 7) had lower logHCCs in the second and third trimesters compared to women without symptoms of anxiety (Supplementary Fig. 2). No differences in the mean of logHCCs were observed with respect to symptoms of antenatal depression and perceived stress (PSS) in the four periods evaluated (Supplementary Fig. 2).

### Multivariable regressions

Parity was excluded from the multivariable analysis as it was strongly correlated with maternal age. Implementing fully adjusted models, we observed that pre-pregnancy BMI, gestational age at HCC, and hair dyeing, were associated with logHCC in different time periods, while difficulty accessing basic foods and symptoms of antenatal depression were associated with logHCC at a single time in pregnancy (Table 4). For instance, a 1 kg/m^2^ increase in pre-pregnancy BMI was on average associated with a 0.02 to 0.03 unit increase in logHCCs (*P* < 0.001) across the four prenatal periods (Table 4). Every 1-week increase in gestational age was associated with a 0.01 unit decrease in logHCC in the first and second trimesters (Table 4), but this association was positive with third trimester logHCC (*β*= 0.04 logHCC units, 95%CI = 0.02, 0.06). Likewise, women who dyed their hair had 0.12 and 0.20 higher logHCC in the first and second trimesters, respectively. Participants with symptoms of antenatal depression (PHQ-9 score ≥ 10) had 0.11 (95%CI= −0.20, −0.02) lower logHCC in the first trimester compared to women with no/mild antenatal depression. Having difficulty accessing basic foods was associated with higher logHCC in the third trimester alone (*β*= 0.18, 95%CI= 0.08, 0.29). Similar associations were obtained in the stepwise adjusted models (Supplementary Table 2).

## DISCUSSION

Pregnancy is one of the most critical periods of human life, requiring massive changes in extremely complex physiological circuits. Interferences in these circuits can have adverse effects across generations. Hence, understanding these changes is key to preventing adverse effects and promoting a healthy life for children. Our study investigated HCC, a biomarker of HPA axis activity, with several covariates, including psychosocial stress measures (stress, anxiety, and depression) during the pre-pregnancy and pregnancy periods in a large sample of pregnant Peruvian women. Overall, we observed increased levels of HCC before and throughout pregnancy, peaking in the third trimester. Furthermore, we found that HCC was associated with pre-pregnancy BMI in all four prenatal periods, but time-specific associations were seen between HCC and gestational age at HCC collection, hair treatment, difficulty accessing basic foods, and symptoms of antenatal depression, after controlling for well-known confounders. We found no statistically significant associations between HCC and symptoms of anxiety or perceived stress neither before nor during pregnancy.

The mean HCC values observed in our sample were, on average, lower compared to other studies that investigated HCC during pregnancy, as shown in a recent systematic review of 56 studies by Marceau et al. ^51^. Nevertheless, the observed mean values in our sample ranging between 3.38 and 5.59 pg/mg, lie within most mean levels observed by Marceau et al. (2020). We are aware of only one study investigating HCC in a pregnant Peruvian sample ^52^ (not overlapping with samples in this study) ^41,44,45,53,54^. Interestingly, Dobernecker et al. ^52^ observed considerably higher HCC levels in 39 participants, which may be due to the small sample size used and the characteristics of the participants, given that more than 50% reported previous experiences of trauma, implicating a higher chronic cortisol secretion. Furthermore, we observed an increase in HCC across pregnancy, peaking in the third trimester. This increase in HCC across pregnancy was also found in previous studies, although not consistently. For example, Marceau et al. ^51^ did not find this increase in half of the reviewed studies, questioning this often-referenced assumption. Recently, this questioning was mitigated, as qualitatively more precise studies examining individual HCC trajectories ^55^ or more fine-grained time intervals ^56^ obtained HCC increases across pregnancy, although in a non-linear way, with interindividual differences and massive within-person variations. This non-linear cortisol increase is in accordance with established biological findings. Beginning with gestational week seven, cortisol secretion is increasingly stimulated by the temporary growth of the pituitary gland and by the placenta ^57–61^, due to an isolated excitatory influence of the placental cortisol-releasing hormone on cortisol secretion, leading to cortisol elevations up to five-fold in certain tissues ^61–64^.

For providing a methodological foundation for further investigations of HCC in pregnant samples, we investigated the bivariate influence of twenty-one pregnancy-related, sociodemographic, and hair-related covariates on HCC. The only covariates that showed a significant bivariate influence on HCC in at least two time points and in a consistent manner were maternal BMI, maternal education, difficulty accessing basic foods, and infant sex, which is partly consistent with previous studies ^38–40,45,65^. For instance, we showed a significant difference in prenatal HCC by infant sex, whereby women giving birth to a female infant had significantly higher HCC preconception and in the first trimester, compared to women giving birth to a male infant. The vast majority of previous studies did not find such an effect ^33,38,39,44,61,66–73^. However, Romero-Gonzalez et al.^65^ recently reported significant differences in prenatal HCC by infant sex. Similar to our findings, they found a higher first-trimester HCC in women carrying a female compared to a male baby ^65^. These findings indicate that prenatal experiences of stress may influence the survival of the fetus and the secondary sex ratio distribution in the population; however, further results from Romero-Gonzalez et al. ^65^ found no significant impact on infant sex by perceived stress.

The correlation observed between educational level and HCC is similarly inconsistent based on results from previous pregnancy studies. While some studies found a statistically significant influence of educational level on HCC ^38–40,74,75^, others found no such effect ^61,68,76–78^. For example, in a subsample of *N* = 62 pregnant women from Spain, HCC differed significantly regarding educational level across the three trimesters, with participants of higher education showing lower HCC in the first and second, but not in the third trimester ^40^. Our results were directionally and time-consistently related to the associations identified by Garcia-Leon *et al*. ^40^. Given the close relationship between educational attainment and income ^79^, it is possible that our finding of the correlation between education and HCC corresponds with the association identified between difficulty accessing basic foods and HCC in our study. This notion is supported by the fact that we found both variables, education and difficulty accessing basic foods, to be correlated, although this correlation was weak. We did not find other studies except our own ^45,53^ investigating difficulty accessing basic foods among pregnant women, which might be explained by the uniqueness of our LMIC sample, as most studies examining HCC among pregnant women have been conducted in high-income countries in North America and Europe ^51^ where including difficulty accessing basic foods might be less important.

In bivariate and multivariable analyses, we found that pre-pregnancy BMI was the strongest predictor of HCC before and during pregnancy. This finding is in accordance with some ^39,44,55,66,74,75,80,81^ but not all ^38,45,67,77,82,83^ previous studies. To our knowledge, only two studies have investigated the association between HCC and maternal BMI across all three trimesters ^40,74^ with partly conflicting findings. While Bosquet Enlow et al. ^74^ found a significant positive correlation between BMI and HCC in the second but not in the first and third trimesters in a sample of *n* = 93 U.S.-American women, Garcia-Leon et al. ^40^ failed to find such an association in their Spanish sample of *n* = 62 women. As we investigated HCC in a pregnant sample three to ten-fold larger than in previous studies, our findings of an unbiased and consistent association between pre-pregnancy BMI and HCC in all four prenatal periods (i.e., from preconception to the third trimester), add to this existing evidence. An association between BMI and HCC is likely, as associations between measures of obesity and both, HPA-axis activity ^84^ and stress ^85^, are well established in non-pregnant samples.

From the different psychosocial stress measures considered in our study, maternal symptoms of antenatal depression was the only measure associated exclusively with first-trimester HCC after controlling for relevant covariates. While such findings have been reported in previous studies, evidence is still conflicting about the temporal aspect of this association. For instance, there have been studies that report a positive association of HCC with antenatal depression in every trimester of pregnancy ^33,34^, except for the pre-pregnancy period ^41^. However, an equal number of studies support a null association of antenatal depression with HCC in late pregnancy ^35–37^, or throughout pregnancy ^41^. Identifying an association of depression with HCC restricted to the first trimester is a plausible finding. One explanation for this finding is the fact that levels of HCC in early pregnancy are lower than in subsequent trimesters, where they tend to be more influenced by physiological factors, which implies that the associations between psychosocial stressors like depression and HCC are more likely to be detected in early versus in late pregnancy ^86^. This observation agrees with the concept of a “ceiling effect” for stressors measured in pregnancy, which suggests that their influence is only apparent when the physiological response measured is low to moderate ^41^. The fact that in our study we assessed antenatal depression at the time of the interview in early and mid-pregnancy and HCC from preconception to the third trimester, indicates that our results are less likely to be influenced by the time in which the stressor was measured and more related to the time in which the psychoendocrine association is interrogated.

Our study has different strengths. First, this is the largest study conducted to date assessing the correlates of HCC in the prenatal period, affording greater power to identify true associations with HCC compared to previous smaller studies. In addition, we were able to assess HCC from preconception through to the third trimester, which allowed us to better characterize HCC’s relative response in relation to multiple factors acting during pregnancy. Lastly, we included a comprehensive list of sociodemographic, pregnancy-related, hair-related, and psychosocial covariates in the characterization of HCC correlates in pregnancy, and this helped us to confirm existing evidence and provide foundational evidence about the role of multiple stressors on relative changes in HCC during pregnancy.

Our study comes with some limitations. Despite our large sample size, not all individuals in our sample had longitudinal measures of HCC from preconception through to the third trimester, reason why comparisons of HCCs were only possible between consecutive time periods, where paired measures were available. Despite this limitation, groups of samples in our study with at least two consecutive measures of HCC in pregnancy (*n* from 60–1,022) were larger than the number of samples included in a recent study addressing longitudinal changes of HCC in pregnancy (*n* = 98) ^41^. The lack of a full longitudinal assessment of HCC in our study prevented us from interrogating individual patterns of HCC change across pregnancy, and from identifying interindividual trajectories of HCC change, and their association with multiple stressors measured in pregnancy. Future studies addressing this gap are required in larger samples from longitudinal pre-birth cohort studies. Lastly, our study included pregnant women from Perú, and the specific socioeconomic and cultural context of this population means that our findings may not be generalizable to other populations from different socioeconomic backgrounds.

## CONCLUSIONS

The need for valid biomarkers of psychological distress is high, but their identification remains a challenge. Facing this challenge, we investigated the novel biomarker HCC and its association with pregnancy-related, sociodemographic, and hair-related covariates in the pre-pregnancy and pregnancy periods. We found that maternal HCC was significantly and consistently associated with pre-pregnancy BMI across four prenatal periods, while other correlates such as gestational age at HCC collection, hair treatment, difficulty accessing basic foods, and symptoms of antenatal depression, showed time-specific associations with HCC after adjustment for relevant covariates. The results of our study set a foundation for a better understanding of the biological factors involved in the regulation of maternal HPA-axis functioning and its influence on psychosocial distress measures in pregnancy. This understanding is urgently needed to eventually reduce the global burden of prenatal psychological distress on adverse health outcomes for the next generation.

## METHODS

### Study population

Participants in this study were drawn from two cohort studies of the same underlying source population. The first study is the Pregnancy Outcomes, Maternal, and Infant Study (PrOMIS), a prospective cohort study designed to assess the maternal social and behavioral determinants of adverse pregnancy outcomes 42. The second study is the Screening Treatment and Effective Management of Gestational Diabetes Mellitus Study (STEM-GDM), a cross-sectional study designed to assess the prevalence of gestational diabetes among pregnant Peruvian women, with the goal of improving GDM screening and management ^43^. Women recruited in both studies attended prenatal care clinics at the Instituto Nacional Materno Perinatal (INMP) in Lima, Perú. The INMP is Perú’s national reference center for perinatal and neonatal care, operated by the Ministry of Health. Details of each study have been provided before ^42,44^. Briefly, for PrOMIS, recruitment started in February 2012 and ended in November 2015. Participants were enrolled in PrOMIS if they initiated prenatal care in early pregnancy (< 16 gestational weeks) and were followed up until delivery. For the STEM-GDM study, recruitment started in February 2013 and ended in June 2014. Participants were invited to participate in the study if they initiated prenatal care prior to 28 weeks (mean gestational age 26 ± 1.3 weeks). For both studies, eligible participants were > 18 years of age, had singletons, were able to speak, read and write in Spanish, and were planning to deliver at the INMP; otherwise, women were excluded. In addition, for STEM-GDM, women were excluded if they had pre-existing diabetes, took glucose-lowering medication, or had a chronic condition ^43^. Human subjects research was conducted according to the principles of the Declaration of Helsinki. Written informed consent was obtained from all participants in PrOMIS and STEM-GDM, and review boards from the INMP and the Harvard T.H. Chan School of Public Health approved all study procedures.

### Analytic sample

**Figure 1:**
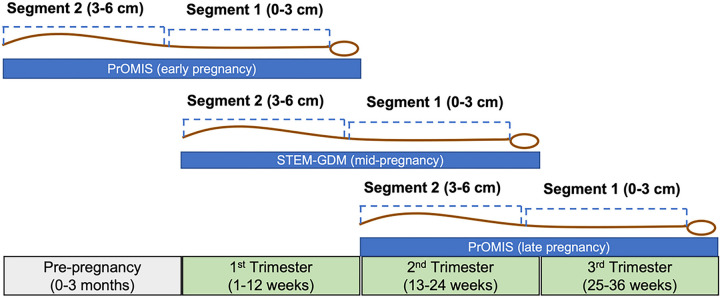
Diagram showing the segmental analysis of HCC from scalp hair samples collected across pregnancy from two pre-birth cohorts in Lima, Perú (N=2,581). Each hair sample collected in early, mid-, and late pregnancy, was segmented into two hair segments (3 cm each) to assess current HCC measures from segment 1 (0–3 cm from the scalp), and HCC in the 3 months prior to the hair sampling from segment 2 (3–6 cm from the scalp).

**Figure 2:**
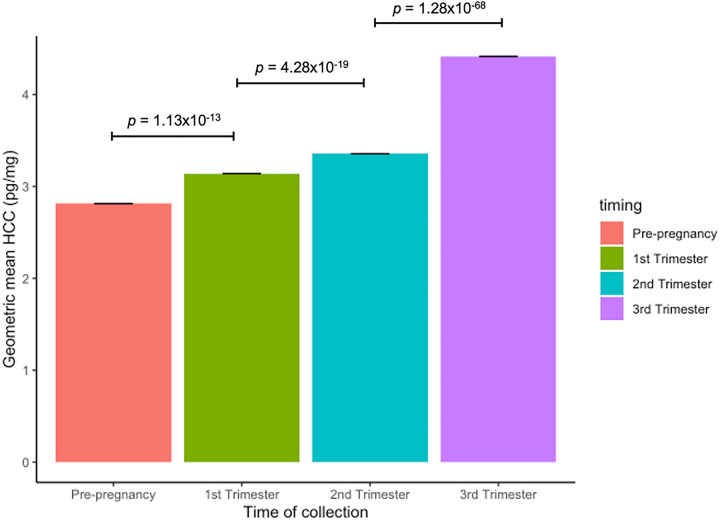
Geometric means of HCC across four prenatal time periods (pre-pregnancy, first trimester, second trimester, and third trimester) in pregnant women in Lima, Perú (N= 2,581). HCC is represented using the geometric mean (pg/mg). *P*-values comparing the statistical difference in the mean of logHCCs across consecutive time periods, were calculated using paired t-tests.

**Figure 3:**
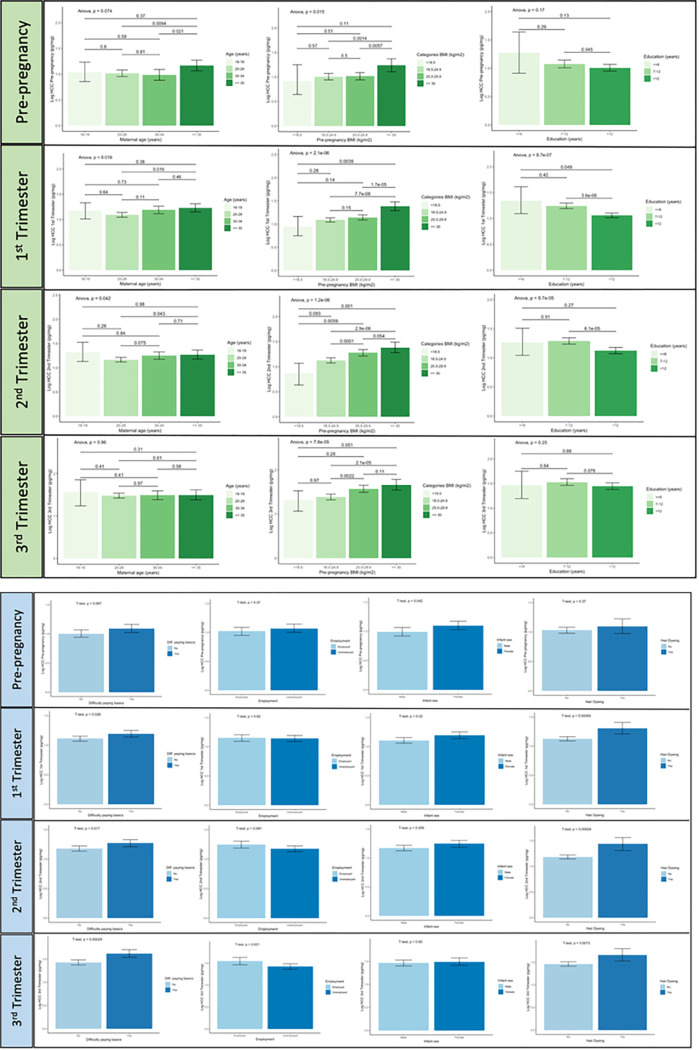
Distribution of Log-transformed values of HCC (logHCC) across categorical variables (N range 835–1734). *P*-values assessing the mean difference in logHCC between groups were calculated using unpaired t-tests for binary variables, and one-way Analysis of Variance (ANOVA) for ordinal variables. The post-hoc Tukey-Kramer test was implemented to report the *p*-value for the pairwise comparison of means across categories of maternal age (years), pre-pregnancy BMI (kg/m^2^), and education (years). Results were considered significant at *p* < 0.05.

Participants included in the analysis were a subset of the total sample in PrOMIS and STEM-GDM who provided a 6 cm scalp hair sample at the time of enrollment. In PrOMIS, 1,623 participants had HCC measured from a hair sample collected at enrollment in early pregnancy (4–19 gestational weeks), while 427 (29%) participants had HCC from a hair sample collected in late pregnancy (24–36 gestational weeks). For STEM-GDM, HCC was available in 530 participants who contributed a hair sample at the time of enrollment in mid-pregnancy (24–28 gestational weeks). Each hair sample was cut into two 3 cm hair segments to assess HCC at two time periods. The hair segment closest to the scalp (0–3 cm) measured HCC around the time of the visit, which occurred either in the first, second or third trimester (Fig. 1). The second hair segment farther from the scalp (3–6 cm) in the same participant, measured HCC retrospectively 3 months prior to the time of hair collection (i.e., HCC pre-conception, in the first trimester or the second trimester, respectively) (Fig. 1). Before analyses, we set as missing values of HCC for 7 participants whose measurements were > 60 pg/mg pre-pregnancy or in the first trimester, > 80 pg/mg in the second trimester, or > 40 pg/mg in the third trimester. These cut-offs corresponded to HCC values that fell more than 3 times outside the interquartile range value of HCC from above (Quartile 3 + 3*IQR) or below (Quartile 1–3*IQR). In total, 2,581 participants were included in the analysis considering their availability of HCC in at least one time period: pre-pregnancy (0–3 months preconception), first trimester (1–12 gestational weeks), second trimester (13–24 gestational weeks), or third trimester (25–36 gestational weeks).

### Hair collection and HCC assessment

Detail of the procedures implemented for hair collection and HCC assessment have been previously described ^41,44,45^. Briefly, trained staff collected a lock of hair (~ 100 strands of hair) from the back of the head (posterior vertex) as close to the scalp as possible. Hair samples were stored in foil paper at room temperature until their analysis. For each participant, each 6 cm of hair was segmented into two 3 cm hair segments and analyzed in the same batch to avoid within-subject variability due to batch effects. Cortisol concentrations were measured in pg/mg and obtained using a standardized liquid chromatography tandem mass spectrometry (LC-MS/MS) assay as previously described ^41,45^, with a lower limit of detection of 0.1 pg/mg. Six blinded control samples were randomly allocated to assess variability ^45^. The inter-assay coefficient of variation was 8.1%, which is within acceptable limits^46^.

### Sociodemographic, pregnancy-related, and hair-characteristic measures

We recorded information about the participant’s sociodemographic and lifestyle variables, anthropometrics, reproductive health, and hair characteristics using structured questionnaires conducted by research staff at enrollment. Sociodemographic and lifestyle variables included age (years), years of education (< 6 years, 7–12 years, > 12 years), ethnicity (mestizo/not mestizo), employment status during pregnancy, marital status, infant sex, difficulty accessing basic foods (yes vs no), smoking, and alcohol use during pregnancy. Anthropometric variables included self-reported pre-pregnancy BMI (kg/m^2^) and infant birth weight (g) abstracted from medical records. Pre-pregnancy BMI was also used categorically (< 18.5, 18.5–24.9, 25–29.9, ≥ 30). Questions about the women’s reproductive health assessed the number of previous pregnancies (parity), if the pregnancy was planned, gestational age at the time of hair collection (weeks), and gestational age at delivery (weeks). Gestational age at visit was calculated using the last menstrual period. Hair characteristics included the natural hair structure (straight, curly), hair color (black, brown, other), frequency of hair washing (1–2, 3–5, or 6–7 times per week), and cutting (every month, every 3 months, 6 months or once a year), the product used to wash hair (shampoo, and conditioner, other), and the use of chemical products for hair tinting and dyeing.

### Antenatal symptoms of Depression, Generalized Anxiety, and Perceived Stress

Different psychosocial stress measures were assessed at the time of the interview in early pregnancy and in mid-pregnancy from participants of the PrOMIS and STEM-GDM studies, respectively, using the same instruments. These scales have been previously translated and validated to use in Spanish-speaking populations ^41^. Symptoms of antenatal depression, perceived stress, and generalized anxiety were assessed using the 9-item Patient Health Questionnaire (PHQ-9) ^47^, the 14-item Perceived Stress Scale (PSS) ^48^, and the 7-item Generalized Anxiety Disorder Assessment (GAD-7) ^49^, respectively. The PHQ-9 measured symptoms of antenatal depression during the last 14 days from the time of the interview, it ranges from 0–27, and a score ≥ 10 was deemed positive for antenatal depressive symptoms ^45,50^. The PSS assessed perceived feelings and thoughts of stress for situations happening during the last month. PSS ranges from 0–56, and was categorized using tertiles, each tertile representing increasing values of the score (Tertile 1 = low, Tertile 2 = middle, and Tertile 3 = high PSS). The GAD-7 scale measured symptoms of antenatal anxiety during the last 14 days, it ranges from 0–21, and a cut-off of 7 was used to define anxiety symptoms.

### Statistical Analysis

Continuous variables were visually inspected for normality using histograms and quantile (Q-Q) plots. Baseline characteristics of participants were described using mean ± standard deviation (SD), or frequency and percent. Due to the right-skewed distribution of HCC,, we used geometric means and SD to compare HCCs concentrations across categories of maternal covariates. Values of HCC were log-transformed (logHCC) for further analyses (Supplementary Fig. 1). We used paired t-tests to compare mean differences in logHCCs for consecutive time periods (i.e., pre-pregnancy vs first trimester, first trimester vs second trimester, and second trimester vs third trimester). Unpaired t-tests and one-way analysis of variance (ANOVA) were used to assess mean differences in logHCCs across categorical binary and ordinal covariates, respectively. Post-hoc Tukey Kramer tests were used in the pairwise comparison of means of logHCCs across categories of ordinal covariates. Spearman correlations were used to evaluate the unadjusted and adjusted correlations of logHCCs with most of the continuous covariates, except for maternal age, pre-pregnancy BMI (log-transformed), and PSS, for which the Pearson method was used. Adjusted correlations were obtained using the residuals from stepwise fitted regressions of logHCCs with the covariates. Multivariable linear regressions were used to estimate the independent associations of logHCCs with multiple covariates and with measures of maternal psychosocial stress using a fully adjusted model. We also used stepwise fitted models to establish the covariates that were more correlated with logHCCs. Covariates included in the stepwise-adjusted and in the fully-adjusted models, were selected based on their association with HCC in at least two time periods from bivariate analyses, and based on previous literature ^11,12^. Thus, adjustment was applied for maternal age, gestational age at HCC collection, pre-pregnancy BMI, difficulty accessing basic foods, education, hair dyeing, and infant sex. Parity was strongly correlated with maternal age (r = 0.5, *P* < 0.01); thus, it was excluded from the final list of covariates to avoid collinearity issues. We reported the coefficients of association (β) and the 95% confidence intervals in logHCC units. To ease interpretation, we also presented association estimates in pg/mg of HCC. For all the analyses, a two-tailed *P* < 0.05 was considered statistically significant. All analyses were conducted in R version 4.1.2.

## Figures and Tables

**Table 1:** Characteristics of narticinants at enrollment N = 2581)

Characteristics	Mean or *N*	*SD* or %
Sociodemographics		
Maternal Age (years) Mean ± SD	28.4	6.27
Maternal Age categorical		
*18–19*	128	5.0
*20–29*	1390	53.9
*30–34*	574	22.2
*≥35*	488	18.9
Education (years)		
*≤6*	58	2.2
*7–12*	1203	46.6
*>12*	1307	50.6
Ethnicity Mestizo	2169	84.0
Employed during pregnancy, yes %	1207	46.8
Married or living with partner	2146	83.1
Planned pregnancy, yes %	1090	42.2
Difficulty accessing basic foods, yes %	998	38.7
Difficulty paying for medical care, yes %	1390	53.9
Smoking during pregnancy, yes %	51	2.0
Alcohol during pregnancy, yes %	208	8.1
Pre-pregnancy BMI (kg/m^2^) Mean ±SD	25.6	4.06
Pre-pregnancy BMI categorical		
*< 18.5*	34	1.3
*18.5–24.9*	1240	48.0
*25.0–29.9*	938	36.3
*≥ 30*	341	13.2
Gestational age at HCC collection (weeks) Mean ± SD	16.7	8.09
Gestational age at delivery (weeks) Mean ±SD	38.7	1.89
Parity	1.13	1.22
Nulliparous, yes %	1118	43.3
Infant sex, Female %	1089	42.2
Infant Birth Weight (g) Mean ± SD	3400	599
Stress measures		
9-item Patient Health Questionnaire-9 (PHQ-9) score Mean ±SD	6.71	4.65
PHQ-9 ≥ 10, yes %	586	22.7
7-item Generalized Anxiety Disorder (GAD-7) score Mean ±SD	6.58	4.56
GAD ≥ 7, yes %	1225	47.5
Perceived Stress Scale Mean ± SD	19.9	7.38
PSS tertiles		
*Tertile 1 (low)*	844	32.7
*Tertile 2 (middle)*	844	32.7
*Tertile 3 (high)*	844	32.7
Maternal HCC Mean ± SD		
Pre-pregnancy (pg/mg)	3.99	5.61
First Trimester (pg/mg)	4.16	4.49
Second Trimester (pg/mg)	4.59	5.75
Third Trimester (pg/mg)	5.99	7.75
Hair Characteristics		
Natural hair color		
*Black*	1522	59.0
*Brown*	191	7.4
*Other*	798	30.9
Natural hair structure		
*Straight*	1909	74.0
*Curly*	595	23.1
Hair washing frequency (in weeks)		
*1–2 times*	148	5.7
*3–5 times*	1717	66.5
*6–7 times*	645	25.0
Product used for hair washing		
*Shampoo only*	805	31.2
*Shampoo and Conditioner*	1690	65.5
*Other*	15	0.6
Tinting, yes %	1077	41.7
Dyeing, yes %	302	11.7
Frequency haircuts		
*Every month*	134	5.2
*Every 3 months*	662	25.6
*Every 6 months*	745	28.9
*Once a year*	751	29.1
*Other*	201	7.8

**Table 2:** Pairwise correlations between log-transformed values of HCCs (logHCCs) and continuous covariates in pregnant women in Lima, Peru (N = 2,581). Correlations were adjusted for maternal age, gestational age at HCC collection (GA), pre-pregnancy BMI and parity

Model	Maternal age (years) ^†^	GA (weeks)	Pre-pregnancy BMI (kg/m^2^) ^†^	Parity	PSS scale	PHQ-9 score	GAD-7 score
HCC Pre-pregnancy							
Unadjusted	0.047	0.031	0.105***	0.102***	−0.036	−0.049	−0.035
Adj. Age	–	0.032	0.094***	0.078*	−0.033	−0.051	−0.036
Adj. Age and GA	–	–	0.091***	0.076*	−0.035	−0.052	−0.035
Adj. Age, GA, and pre-pregnancy BMI	–	–	–	0.057	−0.035	−0.051	−0.034
Adj. Age, GA, pre-pregnancy BMI and Parity	–	–	–	–	−0.042	−0.064.	−0.046
HCC First Trimester							
Unadjusted	0.038	−0.064*	0.120***	0.022	−0.053.	−0.034	−0.032
Adj. Age	–	−0.063*	0.108***	0.002	−0.051.	−0.034	−0.033
Adj. Age and GA	–	–	0.110***	0.018	−0.048.	−0.041	−0.021
Adj. Age, GA, and pre-pregnancy BMI	–	–	–	4.0E-05	−0.049.	−0.046	−0.026
Adj. Age, GA, pre-pregnancy BMI and Parity	–	–	–	–	−0.051.	−0.047	−0.03
HCC Second Trimester							
Unadjusted	0.034	−0.065*	0.133***	−0.019	−0.015*	−0.001	−0.050.
Adj. Age	–	−0.067*	0.122***	−0.037	−0.014*	0.001	−0.051.
Adj. Age and GA	–	–	0.123***	−0.029	−0.020	−0.005*	−0.039
Adj. Age, GA, and pre-pregnancy BMI	–	–	–	−0.045	−0.020	−0.005*	−0.044
Adj. Age, GA, pre-pregnancy BMI and Parity	–	–	–	–	−0.010	−0.008	−0.027
HCC Third Trimester							
Unadjusted	0.006	0.152***	0.177***	−0.099*	−0.020	0.067.	−0.065
Adj. Age	–	0.152***	0.175***	−0.102**	−0.020	0.068.	−0.066
Adj. Age and GA	–	–	0.182***	−0.075*	−0.005	0.052	−0.029
Adj. Age, GA, and pre-pregnancy BMI	–	–	–	−0.102**	0.001	0.053	−0.031
Adj. Age, GA, pre-pregnancy BMI and Parity	–	–	–	–	0.034	0.046	0.008

Note. GA, gestational age at HCC collection (weeks); PSS, Perceived stress scale; GAD-7, 7-item generalized anxiety score; PHQ-9, 9-item Patient Health questionnaire score.

† Variables log-transformed. GA, GAD-7, PHQ-9, and parity were analyzed using the Spearman method. For other covariates, Pearson's correlations were used. Adjusted correlations were obtained after retrieving logHCC residuals from stepwise adjusted linear regression models using as covariates maternal age, GA, pre-pregnancy BMI, and parity.

* *p* < 0.01

** *p* < 0.01

*** *p* < 0.001.

**Table 3:** Geometric means of HCC (pg/mg) at four time periods (pre-pregnancy, first trimester, second trimester, and third trimester) across categories of maternal ba characteristics (N = 2,581).

	HCC Pre-pregnancy (*N*=1023)				HCC First Trimester (*N*=1734)				HCC Second Trimester (*N*=1534)				HCC Third Trimester (*N*=835)		
Characteristic	*N*	Geometric Mean	*SD*	*p*	*N*	Geometric Mean	*SD*	*p*	*N*	Geometric Mean	*SD*	*p*	*N*	Geometric Mean	*SD*
Maternal Age (years)															
*18–19*	52	2.82	1.91	<.05	101	3.33	2.20	<.01	75	3.84	2.35	.04	27	4.66	2.08
*20–29*	548	2.75	2.12		945	2.98	2.01		826	3.21	1.97		437	4.41	1.91
*30–34*	235	2.66	2.22		369	3.28	2.08		335	3.50	2.00		203	4.48	1.94
*≥35*	188	3.22	2.03		318	3.43	2.00		297	3.51	2.19		168	4.33	2.03
Education (years)															
*≤6*	18	3.69	2.18	.07	31	3.84	1.98	<.01	40	3.76	2.07	<.01	27	4.59	2.08
*7–12*	424	2.93	2.02		753	3.46	1.95		767	3.58	2.03		445	4.52	1.97
*>12*	573	2.70	2.18		938	2.88	2.09		722	3.11	2.04		362	4.28	1.90
Ethnicity															
*Mestizo*	829	2.80	2.12	0.65	1417	3.10	2.04	.13	1319	3.29	2.04	<.01	740	4.34	1.91
*Not Mestizo*	192	2.87	2.13		314	3.32	2.03		213	3.78	2.02		94	5.03	2.16
Employment															
*Employed*	534	2.77	2.13	.55	857	3.14	2.04	.91	662	3.5	2.08	.04	346	4.77	2.04
*Unemployed*	487	2.85	2.10		874	3.13	2.04		871	3.25	2.01		489	4.18	1.86
Marital Status															
*Married*	849	2.84	2.16	.20	1444	3.14	2.05	.93	1277	3.33	2.05	.36	691	4.40	1.96
*Not Married*	171	2.65	1.91		284	3.13	1.99		252	3.48	2.04		141	4.47	1.82
Planned Pregnancy															
*Planned*	433	2.94	2.07	.08	712	3.22	2.02	.19	647	3.39	2.09	.70	373	4.34	2.00
*Unplanned*	585	2.71	2.15		1016	3.08	2.05		879	3.34	2.01		455	4.47	1.90
Difficulty accessing basic foods															
*Yes*	446	2.92	2.12	.13	735	3.30	2.08	.01	542	3.56	2.06	.02	259	4.96	1.67
*No*	573	2.72	2.12		989	3.03	2.01		986	3.25	2.03		5.76	4.19	1.92
Difficulty paying for medical care															
*Yes*	421	3.01	2.09	<.01	774	3.26	2.04	.03	953	3.30	2.01	.30	610	4.31	1.93
*No*	589	2.66	2.13		939	3.03	2.04		568	3.44	2.10		220	4.70	1.99
Smoking during pregnancy															
*Yes*	22	2.30	1.85	.27	34	2.87	1.97	.45	28	3.48	1.90	.77	17	5.52	1.66
*No*	999	2.82	2.12		1601	3.15	2.04		1502	3.35	2.05		816	4.40	1.95
Alcohol during pregnancy															
*Yes*	77	3.11	2.31	.27	127	3.34	2.19	.35	130	3.60	2.25	.30	78	4.59	1.83
*No*	941	2.78	2.10		1601	3.12	2.03		1402	3.33	2.02		756	4.39	1.96
Pre-pregnancy BMI (kg/m^2^)															
*<18.5*	10	2.5	1.7	.03	20	2.64	1.64	<.01	24	2.61	1.79	<.01	14	3.67	1.52
*18.5–24.9*	492	2.70	2.13		833	2.98	2.00		736	3.11	2.02		401	4.02	1.91
*25.0–29.9*	384	2.78	2.09		650	3.13	2.09		545	3.58	2.06		122	5.23	1.91
*≥30*	131	3.35	2.12		219	3.91	2.03		207	3.96	2.06		122	5.23	1.91
Parity categorical															
*0*	480	2.68	2.04	<.01	753	3.17	2.03	<.01	464	3.47	2.06	.26	195	4.93	1.95
*1*	360	2.72	2.21		580	2.91	2.03		484	3.24	2.03		270	4.47	2.02
*2*	131	3.26	2.08		264	3.39	2.05		301	3.49	2.13		168	4.21	1.96
*≥3*	49	3.59	1.94		132	3.51	2.08		280	3.23	1.96		199	4.04	1.81
Nulliparous															
*Yes*	480	2.68	2.04	.06	782	3.16	2.02	.77	627	3.31	2.01	.52	329	4.51	1.91
*No*	540	2.92	2.16		947	3.12	2.06		902	3.39	2.07		503	4.34	1.97
Infant sex															
*Female*	442	2.98	2.11	.02	726	3.30	2.12	.01	638	3.48	2.08	.06	355	4.43	2.03
*Male*	426	2.66	2.10		782	3.00	1.99		747	3.24	2.00		399	4.33	1.88
Depression															
*PHQ-9 < 10*	755	2.86	2.16	.23	1290	3.19	2.07	.07	1215	3.35	2.05	.98	688	4.38	1.93
*PHQ-9 ≥ 10*	263	2.69	1.99		437	2.98	1.94		316	3.35	2.2		146	4.56	2.04
Anxiety															
GAD-7 *<*7	681	2.84	2.10	.48	1075	3.18	2.04	.33	652	3.54	2.11	.01	264	4.93	2.01
GAD-7 *≥*7	337	2.74	2.15		650	3.07	2.05		8.76	3.22	2.00		569	4.19	1.90
PSS tertiles															
*Tertile 1 (low)*	339	2.92	2.08	.84	554	3.24	1.98	.27	498	3.46	2.12	.27	288	4.51	2.02
*Tertile 2 (middle)*	342	2,88	2.25		547	3.17	2.10		493	3.23	2.01		292	4.35	1.91
*Tertile 3 (high)*	318	2.78	2.00		594	3.03	2.03		518	3.40	1.99		245	4.35	1.91
Hair structure															
*Straight*	706	2.82	2.12	.81	1197	3.16	2.06	.58	1188	3.30	2.02	.50	702	4.37	1.94
*Curly*	306	2.78	2.12		508	3.10	2.00		281	3.42	2.12		85	4.67	2.03
Hair color															
*Black*	538	2.81	2.13	.27	956	3.17	2.03	.32	970	3.27	2.00	.11	558	4.19	1.91
*Brown*	61	2.43	2.19		108	2.84	1.92		128	3.23	2.13		82	4.27	1.99
*Other*	412	2.87	2.10		642	3.15	2.08		379	3.56	2.13		153	5.51	2.03
Tinting															
*Yes*	448	2.69	2.05	.10	744	3.25	1.98	.07	619	3.55	2.09	<.01	327	4.59	1.96
*No*	564	2.90	2.17		963	3.06	2.09		857	3.19	2.00		465	4.32	1.95
Dyeing															
*Yes*	127	2.98	2.07	.32	190	3.67	1.98	<.01	172	4.16	2.32	<.01	110	5.37	2.04
*No*	885	2.78	2.13		190	3.67	1.98		1302	3.24	2.00		681	4.29	1.93
Product used for hair washing															
*Shampoo only*	263	3.13	2.21	.02	478	3.32	1.95	.11	531	3.47	2.01	.30	322	4.56	1.98
*Shampoo and Conditioner*	740	2.71	2.08		1219	3.07	2.07		938	3.27	2.06		464	4.34	1.94
*Other*	8	2.31	2.14		9	3.66	2.53		7	3.23	1.64		6	3.77	1.86
Hair washing frequency (in weeks)															
*1–2 times*	37	3.43	1.92	.17	72	3.51	1.79	.14	109	3.77	2.13	.16	73	3.78	2.06
*3–5 times*	753	2.75	2.12		1246	3.08	2.08		949	3.28	2.02		462	4.42	1.93
*6–7 times*	221	2.89	2.13		388	3.27	1.96		418	3.36	2.06		257	4.63	1.96
Frequency haircut															
*Every month*	45	2.83	2.09	.01	86	3.23	1.96	.01	89	3.16	2.00	<.01	48	3.58	1.65
*Every 3 months*	272	2.55	2.25		461	2.86	2.22		384	3.04	2.08		195	4.07	1.90
*Every 6 months*	314	2.79	2.10		516	3.14	1.95		425	3.31	2.00		227	4.59	1.97
*Once a year*	295	2.86	2.01		484	3.30	1.97		446	3.63	2.02		264	4.60	2.02
*Other*	83	3.62	2.06		155	3.49	2.00		117	3.81	2.09		45	5.72	1.92

Mean (SD) corresponds to geometric mean values and standard deviation of geometric means of HCC in pg/mg. PHQ-9, 9-item Patient Health Questionnaire. 7-item Generalized Anxiety Disorder. PSS, Perceived Stress Scale.

**Table 4:** Multivariable linear regressions assessing the association of logHCC with maternal predictors. HCC was measured in four time periods (pre-pregnancy, first trimester second trimester and third trimester) (N range 766–1 636)

	LogHCC Pre-pregnancy^a^		LogHCC First Trimester^a^		LogHCC Second Trimester^a^		LogHCC Third Trimester^a^	
	β	95% CI	β	95% CI	β	95% CI	β	95% CI
Maternal age (years)	0.003	−0.006, 0.012	−0.001	−0.007, 0.006	−0.002	−0.008, 0.004	−0.007	−0.015, 0.001
Gestational age at HCC collection (weeks)	0.011	−0.013, 0.035	−0.008*	−0.016, 0.000	−0.009**	−0.016, −0.003	0.039***	0.016, 0.062
Pre-pregnant BMI (kg/m^2^)	0.015**	0.002, 0.029	0.019***	0.009, 0.029	0.023***	0.0140 0.0325	0.033***	0.021, 0.045
Maternal education (years)								
≤6	Ref	–	Ref	–	Ref	–	Ref	–
7–12	−0.115	−0.503, 0.272	−0.081	−0.372, 0.210	−0.017	−0.261, 0.226	0.007	−0.264, 0.279
>12	−0.161	−0.548, 0.226	−0.241	−0.533, 0.050	−0.169	−0.414, 0.075	−0.057	−0.329, 0.216
Maternal Employment								
Employed	Ref	–	Ref	–	Ref	–	Ref	–
Unemployed	0.052	−0.051, 0.155	−0.007	−0.082, 0.068	−0.062	−0.140, 0.015	−0.090	−0.190, 0.010
Infant sex								
Male	Ref	–	Ref	–	Ref	–	Ref	–
Female	0.097	−0.162, 0.142	0.065	−0.009, 0.140	0.057	−0.020, 0.134	−0.002	−0.100, 0.096
Difficulty accessing basic foods								
No	Ref	–	Ref	–	Ref	–	Ref	–
Yes	0.093	−0.010, 0.197	0.066	−0.010, 0.142	0.066	−0.015, 0.147	0.185***	0.079, 0.291
Hair Dyeing								
No	Ref	–	Ref	–	Ref	–	Ref	–
Yes	−0.010	−0.162, 0.142	0.123*	0.005, 0.241	0.203***	0.081, 0.325	0.094	−0.051, 0.239
PSS tertiles								
Tertile 1 (low)	Ref	–	Ref	–	Ref	–	Ref	–
Tertile 2 (middle)	−0.019	−0.142, 0.199	−0.028	−0.120, 0.064	−0.068	−0.161, 0.026	−0.004	−0.121, 0.112
Tertile 3 (high)	−0.033	−0.158, 0.092	−0.073	−0.164, 0.018	−0.029	−0.123, 0.066	0.004	−0.120, 0.127
GAD-7 ≥ 7								
No anxiety	Ref	–	Ref	–	Ref	–	Ref	–
Anxiety	−0.017	−0.092, 0.126	−0.030	−0.108, 0.048	−0.074	−0.155, 0.008	−0.054	−0.167, 0.059
PHQ-9 ≥ 10								
No depression	Ref.	–	Ref	–	Ref	–	Ref	–
Depression	−0.064	−0.181, 0.053	−0.114*	−0.200, −0.027	−0.041	−0.138, 0.055	0.029	−0.100, 0.157

Note. PHQ-9, 9-item Patient Health Questionnaire. GAD-7, 7-item Generalized Anxiety Disorder. PSS, Perceived Stress Scale.

* p < 0.01

** p < 0.01

*** p < 0.001

a Regression estimates for depression, anxiety and perceived stress were obtained from independent fully adjusted models.

## Data Availability

The data that support the findings of this study are not openly available due to reasons of sensitivity. It will be available from the corresponding author upon reasonable request.
